# Effects of elevated carbon dioxide on male and female behavioural lateralization in a temperate goby

**DOI:** 10.1098/rsos.171550

**Published:** 2018-03-28

**Authors:** Josefin Sundin, Fredrik Jutfelt

**Affiliations:** 1 Department of Neuroscience, Uppsala University, Uppsala, Sweden; 2 Department of Biology, Norwegian University of Science and Technology, Trondheim, Norway

**Keywords:** teleost, climate change, anthropogenic, behaviour, *p*CO_2_, CO_2_ exposure

## Abstract

Behavioural abnormality in fishes has been proposed as a significant consequence of the increasing levels of carbon dioxide occurring in the oceans. Negative effects of elevated CO_2_ have been reported for behaviours such as predator–prey interactions, foraging, hearing and behavioural lateralization. Importantly, the effects vary greatly both within and between species, and some recent studies have shown minimal effects of CO_2_ on behaviour. Whether the effect of CO_2_ also varies between males and females is, however, virtually unexplored. According to resource allocation theory, females are expected to be more sensitive to elevated CO_2_, meaning that non-sex-specific studies may overlook ecologically important differences between the sexes. In this study, we investigated the possible differences between males and females in their response to elevated CO_2_ by performing behavioural lateralization tests in adult temperate two-spotted gobies, *Gobiusculus flavescens*. We found that the strength of the side bias (absolute lateralization) was unaffected by the CO_2_ treatment, and there was no difference between males and females. The control fish were slightly right-biased in their behavioural asymmetry (mean relative lateralization of 14). Exposure to high CO_2_ affected this pattern, such that treated fish were slightly left-biased (mean relative lateralization of −10), regardless of their sex. The same results were obtained yet again when the study was repeated during a second year. We discuss our results in light of the great variation in lateralization that has been reported to depend on variables such as species, ecological settings and environmental factors.

## 1. Background

The present dramatic anthropogenic release of carbon dioxide is altering the composition of the atmosphere and consequently that of the dissolved gases in the sea, a process termed ocean acidification [1,2]. Compared to current-day levels (*p*CO_2_ 400 µatm), the ocean *p*CO_2_ may increase to over 900 µatm (corresponding to a pH reduction of 0.3–0.5 pH units) by the year 2100 [3] according to business-as-usual scenarios (i.e. *p*CO_2_ 1000 µatm). The effects on marine organisms of this environmental stressor have been extensively studied, with more than 300 publications per year since 2005 [4]. Through this research effort, we have gained knowledge on the variability in responses to elevated CO_2_, and how behavioural and physiological effects differ between and within species, depending on life stage and ecological circumstances, whether acclimatization times are relatively short or lifelong, and whether patterns observed may shift across exposure times (reviewed in [5,6]). However, even though the effects of this important environmental stressor have been studied in a range of traits and behaviours, sex-specific alterations remain largely unexplored [7]. In fact, the possible effects of sex on responses to elevated CO_2_ have been investigated in fewer than 4% of the ocean acidification literature [7], despite the fact that many other environmental stressors have sex-specific effects [8–10], and hence similar patterns can be expected for elevated CO_2_.

One of the behavioural attributes where treatment effects of *p*CO_2_ in fish have been close to consistently reported is behavioural lateralization, i.e. the asymmetric expression of cognitive functions. Cerebral lateralization (i.e. asymmetric control of cognitive functions) can be detected in the behaviour of an animal by investigating behavioural lateralization under the assumption that behavioural lateralization reflects an underlying asymmetry in the functioning of the nervous system [11]. Several possible advantages of cerebral lateralization have been suggested, such as benefits associated with enabling multiple stimuli to be processed simultaneously [12]. Cerebral laterality may also present disadvantages, for example interference with exploratory behaviour [13]. In fish, the right brain hemisphere mainly processes visual information from the left field of view and vice versa. Therefore, a particular side may be predominantly used in specific situations, leading to behavioural lateralization [11]. The right eye system has been suggested to be used during routine decision-making, while the left eye system is more often associated with investigating novelty [14]. Hence, lateralized individuals tend to respond differently to stimuli depending on whether viewed with the left or right eye, which may create a disadvantage when, for example, a predator appears at random in the environment [12]. CO_2_ exposure has been reported to affect lateralization in the majority of studies investigating behavioural lateralization, for example, by reversing the side bias [15], or by altering the strength of the bias [16]. Overall, however, the direction of the CO_2_ effect is not consistent [15–22], and some studies report no effect of CO_2_ [23,24].

Differences between males and females in behavioural lateralization are known in many vertebrates. For example, in humans and certain species of fish (e.g. zebrafish, *Danio rerio*), males appear to be more strongly lateralized than females ([25,26], respectively). Although several studies on fish found no sex difference (reviewed in [11]), this might be condition-dependent. For example, life stage and/or reproductive state could be an important factor, and fish showing no difference between the sexes in lateralization during non-reproductive times might be lateralized during reproductively active stages, which could be important from a life-history perspective. Differences between males and females in lateralization have been observed when fish were subjected to various stimuli. For example, male and female convict cichlids, *Archocentrus nigrofasciatus*, showed differences in lateralization in combination with aggression and emotionally conditioned stimuli [27,28]. Similarly, in feral guppies, *Poecilia reticulata*, there was a difference in lateralization between the sexes when the fish were observing neutral stimuli compared to social stimuli [29]. Further, the physical complexity of the rearing environment affected laterality differently in males and females in the rainbowfish, *Melanotaenia duboulayi* [30]. These results imply that males and females may respond differently to alterations in their environment, changes that might be manifested through differences in lateralization between the sexes. Many previous studies investigating the effects of elevated CO_2_ on lateralization used larval fish where the effect of sex could not be tested [15,16,18]. It has previously been reported that lateralization in male and female freshwater zebrafish (*D. rerio*), was unaffected by CO_2_ treatment; however, whether female and male marine fish exposed to elevated CO_2_ differ in lateralization has, to our knowledge, not been investigated.

In this study, we examined the possible effects of elevated CO_2_ on behavioural lateralization using the two-spotted goby, *Gobiusculus flavescens*. This species inhabits coastal macro algae-dominated habitats with considerable natural temporal and spatial variations in pH, in contrast with the open ocean habitat [31], and may thus be expected to show some degree of tolerance to varying CO_2_ levels [32]. It has, however, been reported that embryonic development and larval phototaxis were affected by CO_2_ (1400 µatm), suggesting that early life stages may be sensitive [33]. While no effects on spawning, clutch size and egg incubation time were reported in that study, the breeding pairs were only exposed for 24–48 h [33], and it is not known whether longer exposure could cause effects. The two-spotted goby exhibits clear sexual dimorphism during the reproductive season (in northern Europe, from May to July) ([34,35], and references therein). We exposed sexually mature male and female two-spotted gobies to elevated and current-day levels of CO_2_ and subsequently performed a behavioural lateralization test (detour test). The experiment was performed when sexual dimorphism was evident, when the most clear sex effect might be possible to observe (May–June). We hypothesized that exposure to CO_2_ would induce a reduction in relative and/or absolute lateralization because this has been reported in the majority of previous studies on the impact of environmental stressors (CO_2_, temperature and hypoxia) on behavioural lateralization [15–18,20–22,36]. Further, based on previous findings that lateralization in general does not differ between the sexes (reviewed in [11]), but may do so when certain components of the environment are altered [27,28,30], we hypothesized that we would find an interaction between sex and CO_2_ treatment in lateralization.

## 2. Material and methods

The experiment was performed at the Sven Lovén Centre for Marine Infrastructure, Kristineberg, on the west coast of Sweden, over two years (year one: May to June 2014, year two: June 2017). Sexually mature two-spotted gobies were collected using a beach seine-net pulled by hand in bays of the Gullmar Fjord near Kristineberg (58°15′ N, 11°28′ E). The fish were caught during 3–8 May 2014 and 7–8 June 2017 (at the beginning of the breeding season). In the laboratory, the fish were initially housed in flow-through holding aquaria (year one: for 5–10 days, aquaria size: 62 × 39 × 36 cm; year two: for 2–3 days, aquaria size: 58 × 30 × 36 cm (length × width × height)). The species is a sturdy shallow water schooling fish that quickly acclimatizes to laboratory conditions [33], and in the aquaria they rapidly resumed normal behaviour and feeding. Artificial plants were provided to all fish for shelter. Fish were fed with frozen *Artemia* nauplii, mosquito larvae and mysids ad libitum twice a day. Temperature and salinity followed natural conditions in the area (mean ± s.d., year one: temperature: 10.8°C ± 0.63, salinity 24.4 ± 2.40 PSU; year two: temperature: 14.8°C ± 0.19, salinity 25.5 ± 0.39 PSU, data derived from the continuous monitoring of the flow-through system at the station (http://www.weather.loven.gu.se/kristineberg/en/data.shtml)). The light cycle was set to mimic natural conditions (year one: L16 h : D8 h; year two: L17 h : D7 h).

### 2.1. CO_2_ exposure

Methods of CO_2_ manipulation during the exposure were similar to those described in [24]. The fish were transferred to exposure aquaria (year one: exposure start: 13 May, aquaria size: 38 × 36 × 35 cm (volume: 47.9 l), four aquaria in total, 22–35 fish in each, sexes separated; year two: exposure start: 10 June, aquaria size: 30 × 25 × 30 cm (volume: 22.5 l), eight aquaria in total, 11 females per four aquaria and 13 males per four aquaria). The set-up of header tanks and aquaria followed a randomized block design [37]. The size and number of tanks as well as the number of fish per tank changed slightly between years due to space and tank availability; however, the density remained similar between years (year one: 0.46–0.73 fish per litre, year two: 0.48–0.50 fish per litre), and year was accounted for in the analysis (see Results). After overnight acclimatization to the tanks, the CO_2_ level was gradually increased in half of the male and female tanks until it reached the target level of 1000 µatm. The aquaria had a constant supply of flow-through natural seawater (rate of 2 l min^−1^) from four header tanks (50 l; two per treatment). Each header tank had a flow of 5 l min^−1^ of flow-through seawater taken from a 5 m depth and constant aeration. The target value of 1000 µatm for the CO_2_-treatment header tanks was maintained using pH stat Computers (Aqua Medic, Bissendorf, Germany) connected to solenoid valves regulating the administration of 100% CO_2_ gas (AGA, Sweden). The *p*CO_2_ of all aquaria was measured daily using direct *p*CO_2_ measurements with an infrared CO_2_ probe (GMT 222, Vaisala, Finland) connected to a submerged gas-permeable PTFE probe (Qubit systems, Kingston, Canada) according to [38,39]. Temperature and *p*CO_2_ were measured daily (table 1). Data on salinity levels were derived from the continuous monitoring of the flow-through system at the station (table 1) (http://www.weather.loven.gu.se/kristineberg/en/data.shtml) for the total exposure period (year one: 12–18 days, 13–31 May; year two: 18–19 days, 10–28 June). Alkalinity was measured weekly in year one, using an Eppendorf Bio-218 Photometer (Eppendorf, Hamburg, Germany) according to [40], and pH_NBS_ was measured daily in year two using a Hach Lange HQ40D multimeter. pH_total_ was calculated using the constants of [41] and [42] in CO2calc (Hansen, USGS, USA). Alkalinity and pH are reported as a complement and to allow for easier comparison with other studies; they are not explicitly needed because we used direct *p*CO_2_ measurements. It should be noted that pH_NBS_ measurements are generally not considered adequate to accurately calculate *p*CO_2_ in seawater [43].

**Table 1.** Water chemistry data during the CO_2_ exposure for a four header tanks system (control A and B; high CO_2_ A and B). Temperature and *p*CO_2_ were measured daily; data on salinity levels were derived from the continuous monitoring of the flow-through system at the station; alkalinity was measured weekly during the first year and pH_NBS_ daily during second year. The total pH (pH_tot_) was calculated using CO2calc. The data are presented as means with s.d. in parenthesis.

parameter	treatment/header tank	year one	year two
temperature (°C)	control (A + B)	13.8 (2.38)	14.8 (0.76)
	high CO_2_ (A + B)	13.8 (2.34)	15.0 (0.67)
*p*CO_2_	control A	370.8 (66.39)	438.1 (36.55)
	control B	367.1 (79.56)	449.5 (49.71)
	high CO_2_ A	1050.4 (238.04)	1090.7 (93.81)
	high CO_2_ B	939.5 (99.38)	972.2 (72.23)
salinity (PSU)	all treatments	24.7 (3.90)	27.6 (2.04)
alkalinity	control A	2074.7 (157.9)	—
	control B	2016.2 (167.9)	—
	high CO_2_ A	2009.3 (178.0)	—
	high CO_2_ B	2072.1 (148.4)	—
pH	control A	8.11 (0.07) _tot_	8.12 (0.15) _NBS_
	control B	8.15 (0.09) _tot_	8.12 (0.15) _NBS_
	high CO_2_ A	7.68 (0.02) _tot_	7.72 (0.15) _NBS_
	high CO_2_ B	7.69 (0.04) _tot_	7.77 (0.16) _NBS_

### 2.2. Experimental design

The behavioural lateralization tests were carried out under the same environmental conditions (temperature, salinity, light, *p*CO_2_) as the fish had experienced during the exposure (water from the respective header tank was used in the lateralization arena). Fishes were carefully hand-netted from their home tank and individually introduced into a double T-chamber (dimensions: 50 cm long with a 9 cm wide double T-channel (PlastKapTek, Partille, Sweden) [17], water depth: 6 cm). The fish were given 1 min to acclimatize in one of the end sections of the lateralization arena (starting direction was determined by flipping a coin), after which they were gently encouraged to move forwards until a left or right turning choice was made (without touching the fish, using plastic rods in year one and small green nets in year two). For each individual, 10 consecutive runs in alternating order of swimming direction in the double T-chamber was performed to account for any possible asymmetry in the set-up [44]. Water was changed in between approximately every 10th fish, and there was no effect of testing order within each water change (see electronic supplementary material for details). Likewise, there was no effect of testing order within each exposure tank (see electronic supplementary material for details). Turning choices were recorded by direct visual observation (in year one by two observers (JS and JR), in year two by one observer (JS)) and were performed blind regarding CO_2_ treatment in year two (an assistant fetched fish and water (fish were transported between the rooms in a bucket with water from the respective exposure tank) and the lateralization experiment was performed in a different room than the fish holding room; this was unfortunately not possible in year one; see electronic supplementary material for details). There was no effect of experimenter in year one (two sample *t*-tests relative lateralization: *t* = 0.68, *p* = 0.496, absolute lateralization: *t* = −0.38, *p* = 0.703). Owing to logistical constraints in year one, the tests were performed on exposure day 12 for females (25 May 2014) and on exposure day 18 for males (31 May 2014). In year two, all fish were tested after being exposed for 18–19 days. Previous studies report CO_2_ effects on phototaxis behaviour in larvae of two-spotted goby after approximately 10 days of CO_2_ exposure [33], and on behavioural lateralization in adult three-spined stickleback after 20 days of CO_2_ exposure [17], suggesting that an exposure duration of 18 days can be sufficient to detect a behavioural response. Additionally, no effect of exposure duration on effect size was reported in a meta-analysis of several taxa, including fish, [45], suggesting that the difference in exposure time between males and females in year one may be inconsequential (and year was accounted for in the analysis; see Results). All fish that swam the 10 consecutive runs were used in the statistical analyses (*n* year one = females control: 30, females CO_2_: 24, males control: 11, males CO_2_: 19; *n* year two = females control: 16, females CO_2_: 22, males control: 23, males CO_2_: 25). Fish that did not swim along the T-maze for all 10 runs were excluded (*n* excluded fish year one = females control: 5, females CO_2_: 2, males control: 11, males CO_2_: 5; *n* excluded fish year two = females control: 0, females CO_2_: 1, males control: 2, males CO_2_: 2). Even though the number of excluded fish in year one was rather high, we considered excluding individuals not performing well in the lateralization arena a better option than to force the fish to swim all 10 runs, most probably producing unreliable results of lateralization. After the experiment, the fish were weighed (mean ± s.d., female control: 0.73 ± 0.13; female CO_2_: 0.77 ± 0.17; male control: 0.82 ± 0.23; male CO_2_: 0.86 ± 0.14) and thereafter released back into the wild approximately at the site of capture.

### 2.3. Data analysis

Given that the experimental design of the detour test (choice of turning left or right) generates data with a binomial error distribution rather than Gaussian, we analysed the data using generalized linear models (GLMs) with logit-link function and binomial error distribution. For the analysis of turning direction bias, the number of turns to the left over the total number of left and right turns (i.e. 10) was the response variable (corresponding to the relative lateralization index; see below). When a significant effect on turning direction was found, Wilcoxon-signed rank tests against an expected value of 0 were used to clarify whether the fish were significantly left- or right-biased. For the analysis of the strength of bias (irrespective of direction, corresponding to the absolute lateralization index, see below), the response variable was the maximum number of turns to the preferred side over the total number of left and right turns (i.e. 10). For all GLMs, CO_2_ treatment, sex, year, and the two- and three-way interactions were entered as fixed factors. To allow for comparison between previous studies on lateralization and CO_2,_ we also calculated the relative (*L*_R_) and absolute (*L*_A_) lateralization indexes using the formulas: *L*_R_= [(turns to the right − turns to the left)/(turns to the right + turns to the left)] × 100, with values ranging from −100 to +100 (positive values indicating a preference for turns to the right, negative values, a preference for turns to the left), and *L*_A_ = |*L*_R_|, with values ranging from 0 to 100 [46]. Two-way ANOVAs were used for the analyses with relative and absolute lateralization as the response variables, with the same fixed effects as for the GLMs. For all models, non-significant variables were dropped using stepwise backward exclusion starting with the least significant interaction followed by the least significant main effect (*p* > 0.1). Dropped interactions and main effects were again included in the final model, one at a time, to verify that they did not have a significant effect. We used JMP 11 (SAS Institute Inc., Cary, NC, USA) for the statistical analyses.

## 3. Results

**Figure 1.**
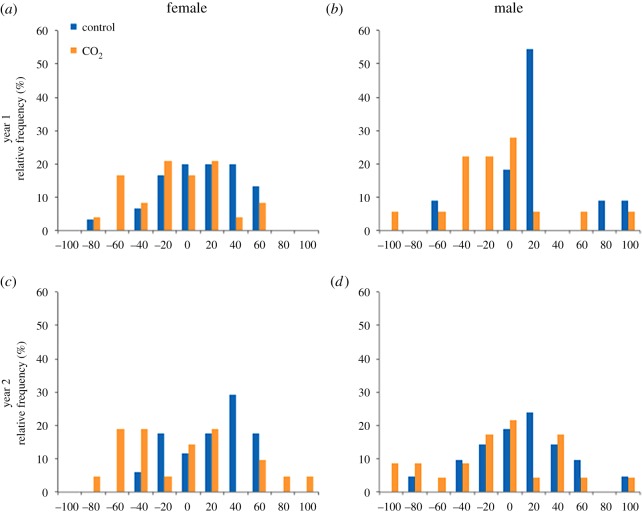
Frequency distribution of the turning direction bias (relative lateralization) in females (*a*,*c*) and males (*b*,*d*) after exposure to control water (approx. 400 µatm; blue bars) or high CO_2_ water (approx. 1000 µatm; orange bars) for year one (*a*,*b*) and year two (*c*,*d*). Individuals with a negative score were behaviourally left-biased, and individuals with a positive score were right-biased.

**Figure 2.**
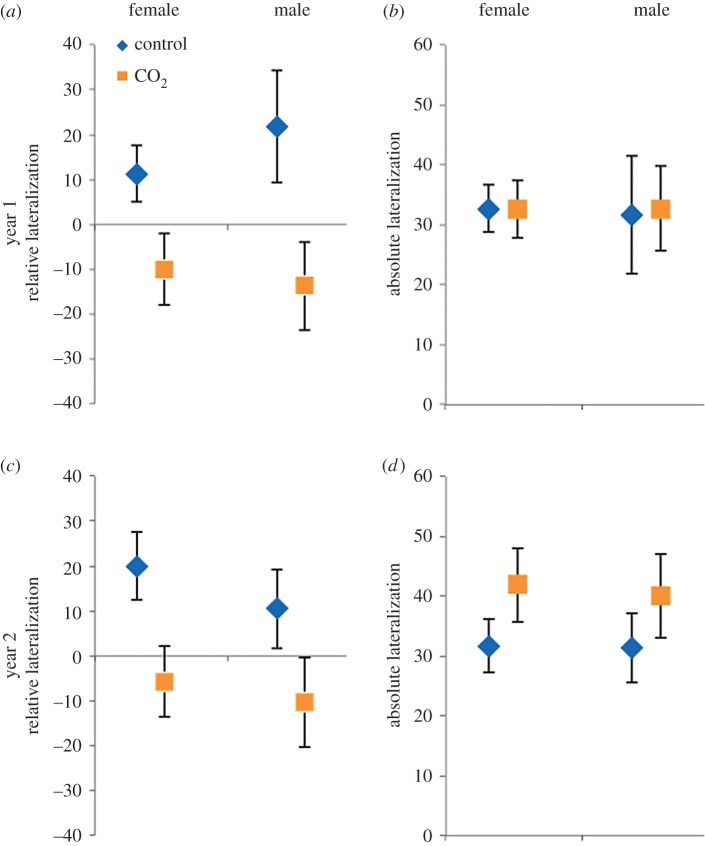
Turning direction bias (relative lateralization) (*a*,*c*) and strength of the turning bias (absolute lateralization) (*b,d*) shown as mean ± s.e. in females and males after exposure to control water (approx. 400 µatm; blue bars and diamonds) or high CO_2_ water (approx. 1000 µatm; orange bars and squares) for year one (*a*,*b*) and year two (*c*,*d*). For the turning direction bias, individuals with a negative score were behaviourally left-biased, and individuals with a positive score were right-biased. For the strength of the bias, higher scores indicate stronger lateralization.

There was a significant effect of CO_2_ treatment on the turning direction bias (table 2, figures 1 and 2). Fish from the control treatment were slightly right-biased (relative lateralization mean ± s.e., 14.4 ± 4.06, Wilcoxon-signed rank against expected value of 0: *W*_78_ = 512.0, *p* < 0.001), while fish from the CO_2_ treatment were slightly left-biased (relative lateralization mean ± s.e., −9.9 ± 4.90, W_86_ = −353.5, *p* = 0.036). There was, however, no effect of sex, and all two-way interactions and the three-way interaction were non-significant (table 2, figures 1 and 2). The ANOVA on the relative lateralization index produced similar results, with a significant CO_2_ treatment effect (*L*_R_ index CO_2_ treatment: *F*_1,164 _= 14.26, *p* < 0.001), and all other fixed effects and interactions non-significant (data not shown). In contrast to the results on the turning direction bias, there was no effect of the CO_2_ treatment on the strength of the bias (table 2, figure 2). Again, there was no effect of sex or of any of the interactions (table 2, figure 2). The ANOVA on the *L*_A_ index produced similar results, with no significant effect of the CO_2_ treatment (*L*_A_ index CO_2_ treatment: *F*_1,164 _= 2.05, *p* = 0.154), or any of the other fixed effects and interactions (data not shown). Including exposure tank as a random effect gave equivalent results, with a significant CO_2_ treatment effect on the turning direction bias, and no significant effects on the strength of the bias (electronic supplementary material, table S1).

**Table 2.** The effect of treatment (high CO_2_ and control), sex, year (2014 and 2017) and their interactions, on turning direction bias (left over total, corresponding to the relative lateralization index) and on the strength of the bias (irrespective of direction, max over total, corresponding to the absolute lateralization index). Parameter estimates are from GLMs and are given as contrasts to the intercept.

response variable	explanatory variable	estimate (± s.e.)	d.f.	*χ*^2^	*p*-value
turning direction bias	intercept	−0.05 (0.07)	—	0.50	0.480
	treatment (CO_2_)	0.24 (0.07)	1, 164	14.11	<0.001
	sex (female)	−0.02 (0.07)	1, 163	0.11	0.744
	year (2017, 2014)	−0.05 (0.13)	1, 162	0.15	0.698
	treatment (CO_2_) × sex (female)	−0.02 (0.07)	1, 160	0.07	0.797
	treatment (CO_2_) × year (2017, 2014)	−0.04 (0.14)	1, 159	0.08	0.775
	sex (female) × year (2017, 2014)	−0.10 (0.14)	1, 161	0.47	0.493
	treatment (CO_2_) × sex (female) × year (2017, 2014)	0.10 (0.14)	1, 158	0.50	0.479
strength of bias	intercept	0.70 (0.05)	—	237.60	<0.001
	treatment (CO_2_)	0.07 (0.05)	1, 164	2.04	0.154
	sex (female)	−0.00 (0.05)	1, 162	0.00	0.983
	year (2017–2014)	0.12 (0.09)	1, 163	1.76	0.184
	treatment (CO_2_) × sex (female)	0.01 (0.05)	1, 159	0.07	0.788
	treatment (CO_2_) × year (2017, 2014)	0.08 (0.09)	1, 161	0.77	0.381
	sex (female) × year (2017, 2014)	0.04 (0.10)	1, 160	0.16	0.162
	treatment (CO_2_) × sex (female) × year (2017, 2014)	−0.01 (0.10)	1, 158	0.01	0.940

## 4. Discussion

The research field of ocean acidification has increased in complexity over the past years, leading to an improved understanding of the variable responses depending on differences between and within species, life stages and ecological circumstances (reviewed in [5,6]). However, less than 4% of the studies on ocean acidification account for sex in the statistical analysis, meaning that the scientific literature in this field is overlooking the importance of sex-based variation [7]. In this study, we investigated sex-specific responses to elevated CO_2_ on behavioural lateralization, a trait where effects of elevated CO_2_ have been close to consistently reported [15–22]. While we found no sex-specific effects, there was a treatment effect on the bias in turning direction (relative lateralization). The gobies shifted from a slight right bias in control water to a slight left bias in elevated CO_2_, while the strength of the bias (absolute lateralization) was unaffected. The effect was identical in both years despite minor differences in methodology, indicating that the effect is robust.

The lack of difference between the sexes is in agreement with several previous studies on behavioural lateralization (reviewed in [11]). However, as differences between the sexes have been observed when fish were subjected to altered environmental components [27,28,47], we hypothesized that we would find an interaction between CO_2_ treatment and sex, but this was not found. To our knowledge, only one study has accounted for sex when investigating the effect of CO_2_ on behavioural lateralization, and no sex-specific effect was found [22]. While this study used a freshwater species (zebrafish), and in addition much higher levels of CO_2_ (1600 µatm), it is still a valuable contribution to the field of aquatic acidification, and it adds support to our results suggesting that males and females are not differently affected by elevated CO_2_ in terms of their behavioural lateralization.

The effects of elevated CO_2_ found here are similar to the results reported for a tropical damselfish, *Pomacentrus wardi*, where CO_2_ exposure caused a shift from a right bias to a left bias without altering the strength of the bias [15]. It has been hypothesized that stressed animals predominantly rely on the right hemisphere of the brain, suggesting that the result found here and in [15] might be a stress response to elevated CO_2_ (i.e. reversal from right-turning bias (left brain control) to left-turning bias (right brain control)). As this hypothesis stems from studies on domesticated mammals (cats and rats) as well as primates and humans [48], further studies on fish would be required for validation. In other species of fish, with no side bias in the control group at the population level, CO_2_ exposure has been reported to reduce the strength of the bias [16–18,21]. In the freshwater zebrafish, *D. rerio*, high levels of CO_2_ (1600 µatm) increased the proportion of right turns [22], meaning that the CO_2_ effect on lateralization in this species was the opposite compared to the results found here, and to those reported in tropical damselfish [15]. Many previous studies report mean absolute lateralization values that are higher than those found here, around 40–50 compared to 33.7, and it could be hypothesized that a stressor cannot decrease the absolute lateralization if it is already at low levels. However, Lai *et al*. [20] reported a mean absolute lateralization for control fish of 33.3, and CO_2_-exposed fish had a significantly lowered absolute lateralization (13.8). Hence, even at relatively low absolute values around 30 it is possible to detect a reduction in CO_2_-exposed fish, although the decrease in Lai *et al*. [20] was more likely because of the lower absolute lateralization expectancy given the usage of 20 runs per individual instead of the more commonly used 10 runs. The reason different species may respond differently to environmental disturbances is unknown but could potentially be related to their habitat (e.g. open water or benthic), because of differing temporal and spatial variation in CO_2_ levels [31], or lifestyle (e.g. aerobic swimmer or benthic ambush predator) as their respiratory and acid–base physiology differ [6]. Differences may also be due to osmoregulatory challenge (i.e. hyper- versus hypo-osmotic relative to the water), stress tolerance [32] or phylogeny, if some orders of fish are more tolerant due to evolutionary history [49]. Additionally, because fish can sense the ambient *p*CO_2_ [39,50], this means that if CO_2_ is continuously perceived as a stressor, as previously suggested [39], this perception may cause stress-sensitive species to respond to the treatment, while stress-tolerant species may respond differently or not at all.

In many vertebrates, the right eye system has been suggested to be predominantly used during routine decision-making, while the left eye system is more often associated with investigating novelty [14]. The effect of CO_2_ on relative lateralization found here could therefore be due to stress or an altered state of alertness, causing a shift from a right-eye system familiarity state to a left-eye system novelty state. On the other hand, it has also been suggested that turning preference may mirror an eye preference for the fish to look behind itself. This hypothesis would suggest that when the CO_2_-exposed fish in this experiment increased the proportion of left turns, they tended to respond differently to the plastic rod or net behind them, looking at it with the left eye, suggesting right hemisphere processing [46].

As the effect of CO_2_ treatment was only present in the turning direction bias (relative lateralization), but not in the strength of the side bias (absolute lateralization), the overall importance of this behavioural shift is unknown. The direction of lateralization can change within individuals and/or populations depending on a range of variables and settings, such as time in captivity [46], sexual motivation [44], the physical complexity of the rearing environment [30], predation pressure [51,52] and parasite prevalence [53]. Thus, as the pattern and strength of laterality in fish vary between as well as within individuals, populations and species, and because they are influenced both by behavioural and ecological variables, laterality could be a plastic trait shaped by environmental conditions, which may be altered through individual experience [11,54,55]. Hence, although CO_2_ may shift the side preference of two-spotted gobies through unknown mechanisms, it is not clear if such an effect may have ecological consequences in wild fish. As it has been suggested that the left and right eyes are used for different tasks [14], it is possible that the shift in eye use may have biologically relevant effects. The fact that the responses of control and CO_2_-exposed fish were extremely similar between years, with no effect of year detected in the statistical analysis, furthermore suggests that this behaviour is consistent in the two-spotted goby. Although there was no effect of sex in this study, future research should incorporate the possible effect of sex to realistically predict the consequences of elevated levels of CO_2_.

## 5. Conclusion

We have shown that males and females did not differ in their degree of lateralization nor responded differently to the CO_2_ treatment. The exposure to CO_2_ had an impact on the direction of the side bias, but not in the strength of the bias, a result that was consistent between years. As the direction of side bias in general shows great variation between species, populations and the relative experience of the individual, the importance of relative lateralization on fitness remains unknown [11]. Further, most studies on the costs and benefits of lateralized behaviour merely document the individual correlation between the degree of lateralization and the performance in other behavioural tests. It remains to be shown whether stronger lateralization is the cause or the effect of high performance in other behaviours, or if they are both independently related to a third cognitive function. Therefore, the causal link between lateralization and fitness remains speculative [11,29,56]. Despite the significant change in the turning direction bias after short-term CO_2_ exposure, we cannot predict if these effects would appear after generations of slowly increasing CO_2_ levels, such as during severe ocean acidification.

## Supplementary Material

ESM Goby lat

## Supplementary Material

Raw data

## Data accessibility

The dataset supporting this manuscript is available as electronic supplementary material and is publicly archived in the repository Dryad (https://doi.org/10.5061/dryad.hs4557q) [57], following best practice guidelines [58].
