# Supplementary material for: Effects of elevated carbon dioxide on male and female behavioural lateralization in a temperate goby
Source: R Soc Open Sci. 2018 Mar 28;5(3):171550. doi: 10.1098/rsos.171550 (PMC5882688; doi:10.1098/rsos.171550)
Supplement: ESM Goby lat [file rsos171550supp1.docx]

***Electronic Supplementary Material to the article:***

**Effects of elevated carbon dioxide on male and female behavioural lateralisation in a temperate goby**

Josefin Sundin^1,2*^, Fredrik Jutfelt^2^

^1^Department of Neuroscience, Uppsala University, Sweden

^2^Department of Biology, Norwegian University of Science and Technology, Norway

^*^Corresponding author: Josefin Sundin

josefin@teamsundin.se

**Material and methods**

*Experimental design*

Water change: The water in the lateralisation arena was changed in-between approximately every 10:th fish year one, and in-between each new tested exposure tank in year two (i.e., in-between each 8-14:th fish). There was no effect of testing order within each water change (GLM left over total: χ^2^_1,164_ = 0.23, *p* = 0.630; max over total: χ^2^_1,164_ = 0.20, *p* = 0.659). The water was changed in the lateralisation arena by carefully flipping the arena on its side, pouring out the water. New water (from the respective header tank) was added to the arena from a bucket until a water depth of 6 cm was reached.

Testing order: The fish were not individually tagged each fish was only once, hence, used fish could not be returned to the exposure tank and placed together with unused fish. This led to decreasing density in the exposure tank during testing, which is very common in behavioural experimentation in fish when not tagging the fish. There was, however, no effect of testing order within each exposure tank (GLM left over total: χ^2^_1,164_ = 0.66, *p* = 0.416; max over total: χ^2^_1,164_ = 1.63, *p* = 0.201). This indicates that the density did not have a major impact on the results of the lateralisation experiment.

Transportation of fish: In year one, the lateralisation trials were performed in the same laboratory room as the fish were exposed. The fish were carefully hand-netted from their home tank and introduced into the arena. Air-exposure was minimal and only lasted a few seconds. In year two, the lateralisation trials were performed in a room adjacent to the exposure room to allow blind scoring of the lateralisation test. An assistant transported the fish between the rooms in a bucket with water from the respective exposure tank. The transportation time was less than one minute. The assistant also fetched water from the header tanks to be used in the lateralisation arena.

**Results**

Including tank as a random effect gave equivalent results, with a significant CO_2_ treatment effect on the turning direction bias, and no significant effects on the strength of the bias (Table S1, Generalized Linear Mixed Models with tank as random effect, analysis performed using GenStat 8, VSN International Ltd, Hemel Hempstead, UK).

**Table S1.** The effect of treatment (high CO_2_ and control), sex, year (2014 and 2017) and their interactions, on turning direction bias (left over total, corresponding to the relative lateralisation index) and on the strength of the bias (irrespective of direction, max over total, corresponding to the absolute lateralisation index).

| **Response variable** | **Explanatory variable** | **Elim. num.** | ***Wald χ*^2^** | ***p-value*** |
| --- | --- | --- | --- | --- |
| Turning direction | Treatment | Kept | 14.00 | < 0.001 |
| bias | Sex | 5 | 0.11 | 0.745 |
|  | Year | 6 | 0.10 | 0.746 |
|  | Treatment*Sex | 3 | 0.06 | 0.804 |
|  | Treatment*Year | 2 | 0.05 | 0.818 |
|  | Sex*Year | 4 | 0.47 | 0.493 |
|  | Treatment*Sex*Year | 1 | 0.49 | 0.484 |
| Strength of bias | Treatment | Kept | 2.04 | 0.154 |
|  | Sex | 5 | 0.07 | 0.794 |
|  | Year | 6 | 1.76 | 0.185 |
|  | Treatment*Sex | 2 | 0.00 | 0.944 |
|  | Treatment*Year | 4 | 0.77 | 0.381 |
|  | Sex*Year | 3 | 0.16 | 0.687 |
|  | Treatment*Sex*Year | 1 | 0.01 | 0.940 |
